# A physiotherapists perspective of a geriatric clinic in a tertiary oncology centre

**DOI:** 10.3332/ecancer.2024.1774

**Published:** 2024-09-20

**Authors:** Ankita Chitre, Akhil Kapoor, Bipinesh Sansar, Anuj Gupta, Praveen Lakshmanamurthy, Somnath Dey, Kunal Vinayak, Ajit Sahoo, Navneet Kaur, Sumaiya Azeem, Dipti Kadu, Akash Anand Shrivastav

**Affiliations:** 1 Department of Physiotherapy, Mahamana Pandit Madan Mohan Malaviya Cancer Centre, Varanasi 221005, India; 2 Department of Medical Oncology, Mahamana Pandit Madan Mohan Malaviya Cancer Centre, Varanasi 221005, India; 3 Department of General Medicine, Mahamana Pandit Madan Mohan Malaviya Cancer Centre, Varanasi 221005, India; 4 Department of Pain & Palliative Medicine, Mahamana Pandit Madan Mohan Malaviya Cancer Centre, Varanasi 221005, India; 5 Department of Occupational Therapy, Mahamana Pandit Madan Mohan Malaviya Cancer Centre, Varanasi 221005, India; 6 Department of Dietitics, Mahamana Pandit Madan Mohan Malaviya Cancer Centre, Varanasi 221005, India; 7 Department of Medical Administration, Mahamana Pandit Madan Mohan Malaviya Cancer Centre, Varanasi 221005, India

**Keywords:** geriatrics, elderly, ageing, geriatric oncology

## Abstract

**Aims and objectives:**

To analyse various domains amongst the geriatric population such as age, gender, body mass index (BMI), comorbidities, type of cancer and use of assistive devices, and find a correlation between the outcome measures such as short physical performance battery (SPPB) and performance-oriented mobility assessment (POMA).

**Methodology:**

Patients above 60 years were screened and further referred to the physiotherapy department. A brief history was recorded to retrieve the demographic data such as name, age, gender, height, weight, BMI, hand dominance, diagnosis, previous investigations are done, comorbidities if any present, use of assistive devices if required and in case any previous oncological treatment has been delivered. Various outcome measures were administered such as POMA, SPPB, 6 minutes walk test (6 MWT) and numerical rating for fatigue. The interpretations were noted on a case report sheet and the appropriate interventions for the deficits were delivered to the patient. Also, the patients were asked to carry on the necessary investigation (if required) and get back to the physiotherapy OPD. No follow-up is required by the patients as this was a retrospective single-endpoint study.

**Results and analysis:**

The descriptive analysis was done by using R software (version 4.2.3). The main objective was to analyse the variables descriptively using numbers and percentages. The correlation between 2 outcome measures: SPPB and POMA was assessed using Spearman’s rank correlation.

All the 100 patients had solid tumour malignancies, commonly GI (37%), thoracic (18%), breast (17%), H and N (13%), uro-oncology (11%) and gynecology (4%). The median age was 70 years (range, 60–88). The median BMI was 22.10 (IQR, 19.40–24.77). Among 100 patients, comorbidities were found in most of the patients, most commonly hypertension (35%), diabetes mellitus (20%), heart disease (9%) and other diseases (8%). Out of 100 patients, 15% of them used assistive devices but the remaining 85% of patients did not require any assistive devices. Different outcome measures were also assessed for understanding the patients’ risk in different categories. On assessing POMA, most of the patients had a medium risk of fall (49%), followed by high risk (31%) and low risk (14%). On assessing SPPB, most of the patients had low risk (41%), followed by medium risk (31%) and high risk (28%). The aerobic capacity of patients was assessed using 6 MWT (walking capacity) which showed that most of them had a severe reduction in aerobic capacity (37%) followed by moderation reduction (28%), good aerobic capacity (25%) and mild reduction (10%). The treatment required by the patients involved most commonly LL strengthening (71; 30.6%) and aerobic conditioning (67; 28.9%) and the least was brisk walking (4; 1.72%) and UL strengthening (2; 0.86%).

**Conclusion:**

Commonly deranged domains included fatigue (97%), risk of fall (80%)**,** reduced aerobic capacity (75%) and comorbidities (73%). The correlation between SPPB and POMA was assessed using Spearman’s rank correlation method which obtained a correlation coefficient of 0.79 which implies that there is a strong positive association between SPPB and POMA.

## Background

An age above 60 years is considered to be the young elderly in India. The geriatric population is vulnerable to several disabilities such as risk of falls, balance issues, gait abnormalities and self-independence leading to activity limitation and participation restriction in the community due to a couple of deficits [12]. Therefore, there is a need to focus on this population and work on the attributes that contribute to these vulnerabilities [6]. Geriatric assessment is a multi-disciplinary approach mechanism [9]. In 2023, The National Cancer Institute came up with data stating that geriatric assessment reduces cancer treatment side effects. The physical therapy aspects of a comprehensive geriatric assessment profile of Indian patients is a neglected aspect and it needs more focus [10, 11]. A study done by Vanita *et al* [1] states that the geriatric assessment adds valuable information to the oncologic decision-making process for older persons with cancer. Another study mentions, that there is a need for awareness of geriatric oncology among practicing physicians who care for older patients with cancer [2]. Only a few institutes in India provide specialized geriatric oncology services. Recognising the importance and implementing such a service to optimally manage this vulnerable population is an urgent necessity. There is a need to develop and validate suitable and culturally appropriate tools to assess older Indian patients with cancer [2]. The US National Comprehensive Cancer Network and International Society of Geriatric Oncology recommendation for geriatric oncology assessment is to be a shoulder on the following features: functional status, comorbidity, cognition, mental health status, fatigue, social status and support, nutrition and presence of geriatric syndrome [3].

Taking into consideration, the physical therapy aspects a few outcome measures which met the psychometric properties were selected such as performance-oriented mobility assessment (POMA) [4, 14]. Short physical performance battery (SPPB) [7, 13]. Six minutes walk test (6 MWT) [8, 15] and numerical rating scale for fatigue. The POMA assesses domains such as balance and gait and a total score is 28. A domain of balance has a score of 16 whereas, a domain of gait has a score of 12. The interpretation of scores is less than 19 (high risk of fall), 19–24 (medium risk of fall) and 25–28 (low risk of fall). SPPB assesses domains such as balance and gait speed done via a 4-m walk test and lower limb strength calculated via a chair stand test. Each domain has a score of 4. The total score on the test was 12. A score between 9 and 12 (low risk), 7 to 9 (medium risk) and 6 and below (high risk). There are various studies that compare the SPPB and POMA outcome measures in the elderly [16–18]. The other outcome measure used was 6 MWT which takes into consideration the walking capacity and is used to assess the aerobic capacity of the patient. This also leads to training the patient at 60%–70% of their capacity which will enhance their overall fitness. A numerical rating scale for Fatigue is an 11-point Likert scale, which has the highest score of 10 and the lowest being 0, the higher the score, the higher the level of fatigue. However, there is no study done on elderly oncology patients.

Also, every patient was prescribed a detailed intervention program which included lower limb strengthening, aerobic conditioning, fatigue management strategies, and gait and balance training as per their deficit, and a few patients came to the clinic for a follow-up. Therefore, we aim to describe the various domains from the physical therapy perspective of Indian geriatric oncology patients in a tertiary setup. Also, there could be a future scope to the study where the pre versus post-operative status, in relation to chemotherapy-induced neuropathy could be followed up. Since this was a single-point retrospective study, the said domains were not assessed.

## Methods

The study was a retrospective design that was carried out in the Department of Physiotherapy which included the geriatric population (60 years and above). The method used was via convenient sampling and included 100 patients from December 2021 to April 2023. The inclusion criteria of the study were: patients above the age of 60 years, both genders who were referred from the Department of Medical Oncology and who did not receive any form of treatment such as surgery, chemotherapy or radiotherapy. The outcome measures included: POMA- English Version, SPPB- English Version and 6 MWT.

### Outcome measures used

1. POMA- English Version
2. SPPB- English Version
3. 6 MWT.

### Protocol

- Patients above 60 years were screened and selected by the Department of Medical Oncology. The patients were further referred to the physiotherapy department to assess their physical domains.
- All the patients who visited the physiotherapy department post-referral were assessed and a brief history was recorded to retrieve the demographic data such as name, age, gender, height, weight, body mass index (BMI), hand dominance, diagnosis, previous investigations done, comorbidities if any present, use of assistive devices if required and in case any previous oncological treatment has been delivered.
- Furthermore, the various tests were carried out using therapist-administered outcome measures such as POMA, SPPB, 6 MWT and numerical rating for fatigue. The POMA considers various aspects such as gait and balance abilities. The SPPB assesses domains such as balance using the balance test, the gait is assessed using the 4-m walk test in which the best of the 2 readings is recorded and lower limb strength is assessed using the chair stand test in which the patient is made to perform 5 repetitions of sit to stand from the chair with arms crossed in front of the body and the time duration is recorded. The 6 MWT was used to calculate the aerobic capacity of the elderly which emphasized that the patient would walk for 6 minutes at a pace as fast as they could and in case of fatigue or tiredness, could take a pause and start the test again from the time when it was paused. The walkway of 20 m was adapted and the calculations were done using the constant formula which was different for males and females and also considered the height, weight and age of the elderly. Also, the fatigue was recorded by verbally asking the patient to score the level of fatigue between 0 and 10 (0 being no fatigue and 10 being the highest level of fatigue). The interpretations were noted on a case report sheet and the appropriate interventions as per the deficits were delivered to the patient. Also, the patients were asked to carry on a necessary investigation (if required) and get back to the Physiotherapy OPD. There was no follow-up required by the patients for this particular study due to the retrospective setting.

## Results

**Figure 1.**
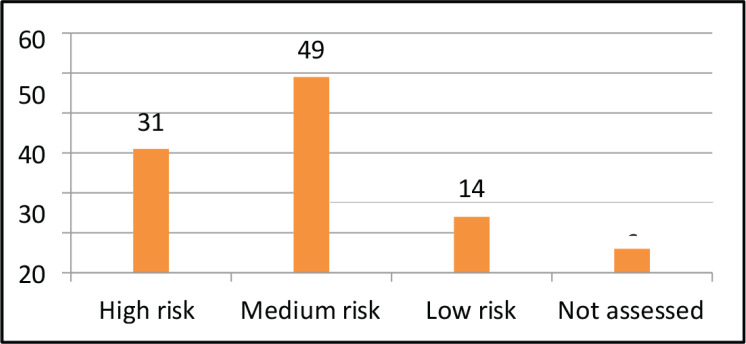
POMA.

**Figure 2.**
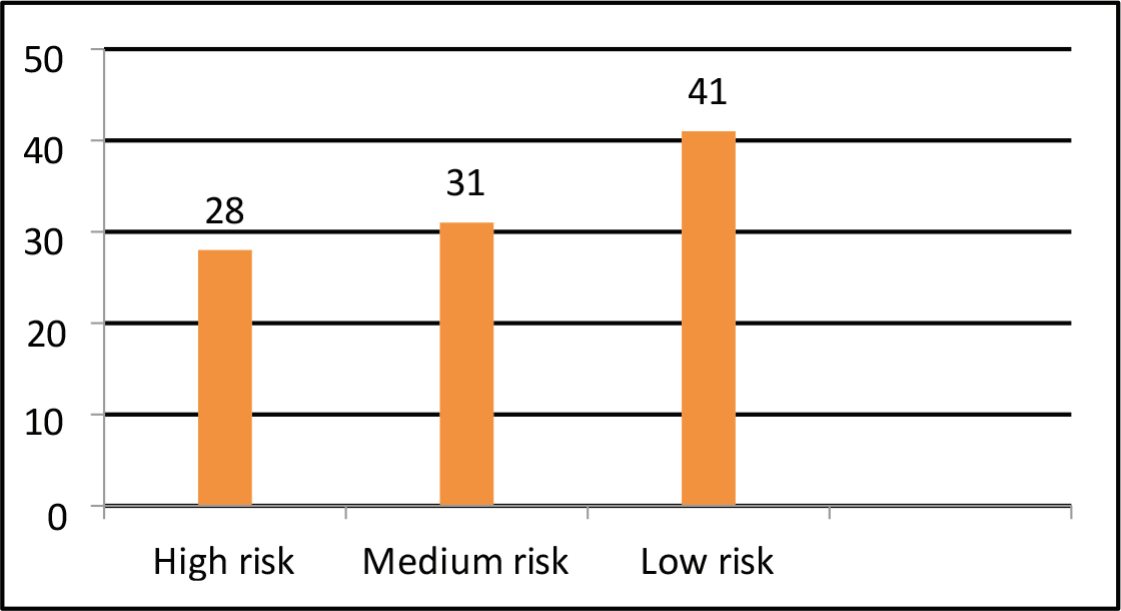
SPPB.

**Figure 3.**
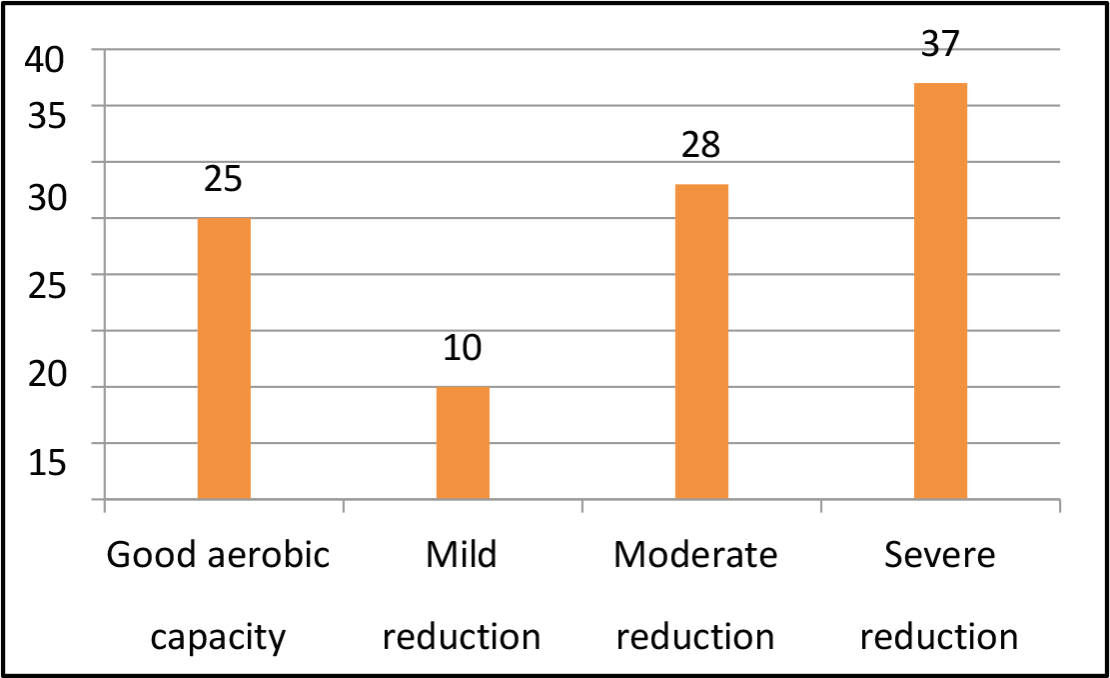
6 MWT.

**Figure 4.**
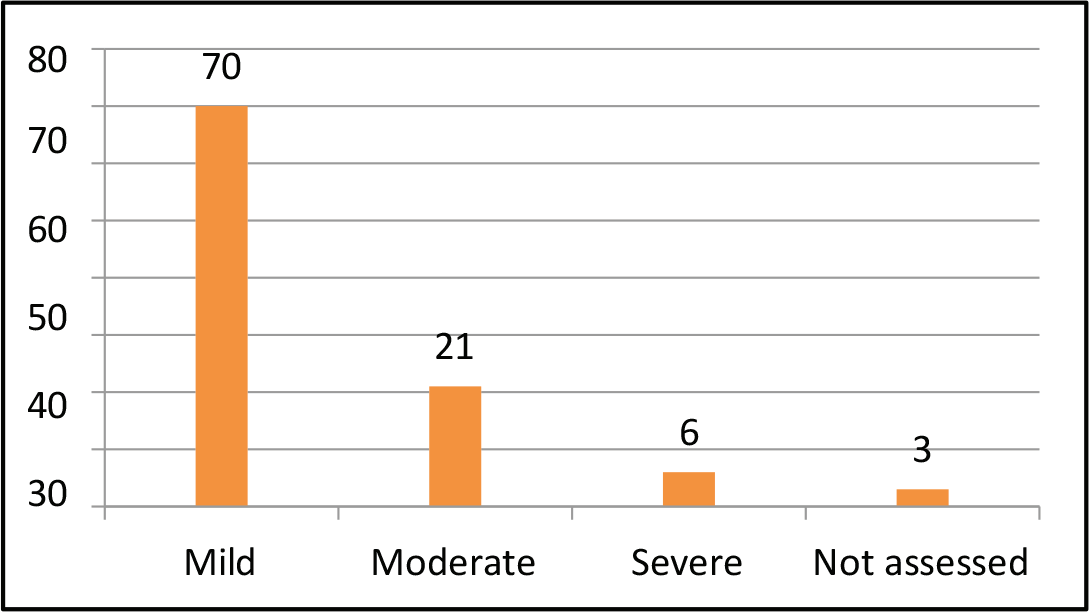
Fatigue (numerical rating scale).

**Figure 5.**
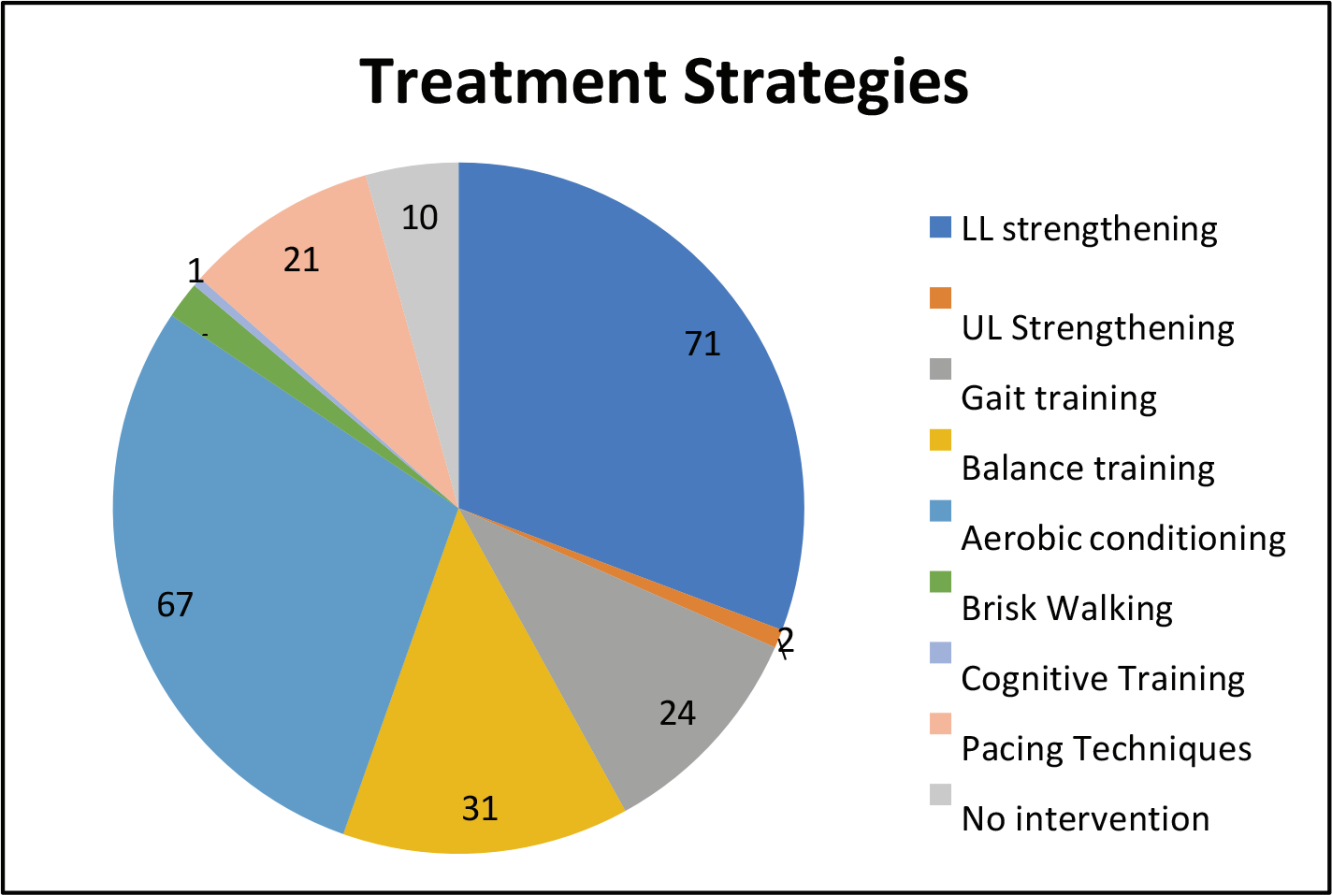
Treatment delivered.

**Figure 6.**
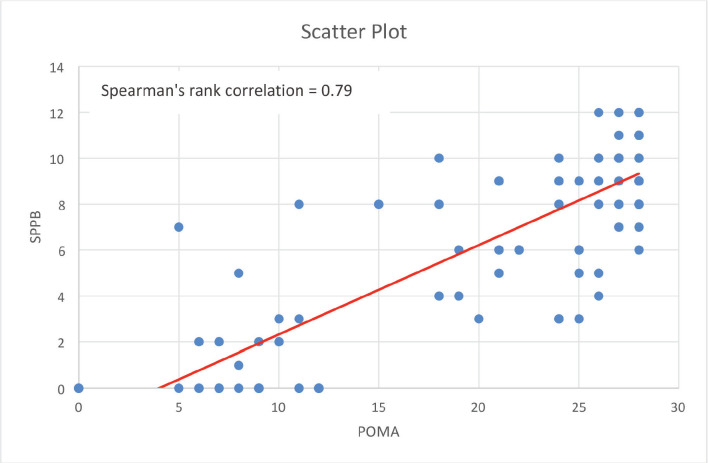
Correlation between POMA and SPPB.

**Figure 7.**
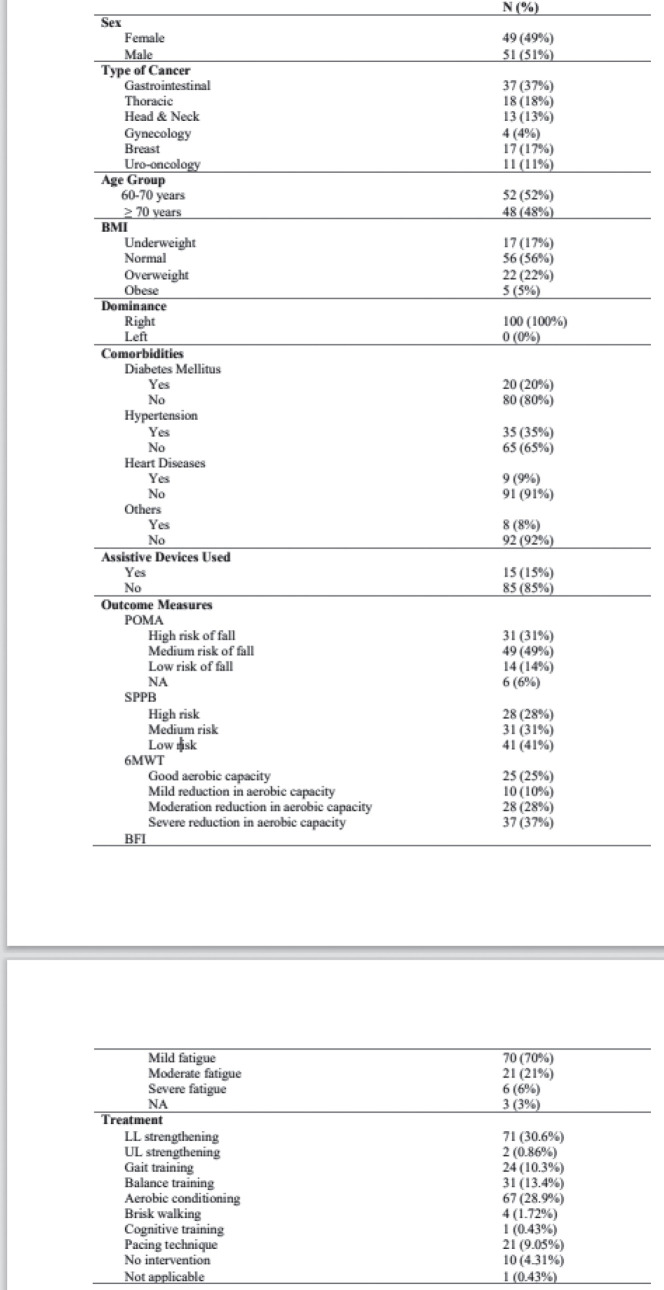
Comprehensive results.

The descriptive analysis was done using R software (version 4.2.3). The main objective was to analyse the variables descriptively using numbers and percentages (see Figures 1–7). The correlation between 2 outcome measures: SPPB and POMA was assessed using Spearman’s rank correlation (Figure 6).

The results of our study are more or less similar to a study done by Vanita *et al* [5] which are comparably stating that in our study, all 100 patients had solid tumour malignancies, commonly GI (37%), thoracic (18%), breast (17%), H and N (13%), uro-oncology (11%) and gynecology (4%). The median age was 70 years (range, 60–88). The median BMI was 22.10 (IQR, 19.40–24.77). Among 100 patients, comorbidities were found in most of the patients, most commonly hypertension (35%), diabetes mellitus (20%), heart disease (9%) and other diseases (8%). Out of 100 patients, 15% of them used assistive devices but the remaining 85% of patients did not require any assistive devices. Different outcome measures were also assessed for understanding the risk of patients in different categories. On assessing POMA, most of the patients had a medium risk of fall (49%), followed by high risk (31%) and low risk (14%). On assessing SPPB, most of the patients had low risk (41%), followed by medium risk (31%) and high risk (28%). The aerobic capacity of patients was assessed using 6 MWT (walking capacity) which showed that most of them had a severe reduction in aerobic capacity (37%), followed by moderate reduction (28%), good aerobic capacity (25%) and mild reduction (10%). The treatment required by the patients involved most commonly LL strengthening (71; 30.6%) and aerobic conditioning (67; 28.9%) and the least was brisk walking (4; 1.72%) followed by UL strengthening (2; 0.86%). Therefore, there is a need for geriatric patients to undergo physical training as per requirements and deficits [19–21].

## Conclusion

Commonly deranged domains included risk of fall (80%)**,** reduced aerobic capacity (75%) and comorbidities (73%). The correlation between SPPB and POMA was assessed using Spearman’s rank correlation method which obtained a correlation coefficient of 0.79 which implies that there is a strong positive association between SPPB and POMA.

## Clinical significance of the study

The results of the study state that, there is a higher risk of fall, reduced aerobic capacity and comorbidities amongst the Indian elderly which need to be addressed early and appropriate interventions at an early stage would prevent the geriatric population from facing debilitating issues and therefore will improve the quality of life and enhance the wellbeing of the elderly patient undergoing oncology treatment.

## Conflicts of interest

There is no conflict of interest from any of the authors.

## Funding

There was no funding source for this particular study.

## Consent

This was a retrospective study. Therefore, no consent was obtained as only the data were analysed.

## Author contributions

The compilation of the demographic data into the Excel sheet, formulation of the study aims and objectives, statistical analysis and design was carried out by the first author. The other co-authors have contributed to the collection of data during routine patient visits to the outpatient department.
